# State and Territorial Laws Prohibiting Sales of Tobacco Products to Persons Aged <21 Years — United States, December 20, 2019

**DOI:** 10.15585/mmwr.mm6907a3

**Published:** 2020-02-21

**Authors:** Kristy Marynak, Margaret Mahoney, Kisha-Ann S. Williams, Michael A. Tynan, Elizabeth Reimels, Brian A. King

**Affiliations:** 1 Office on Smoking and Health, National Center for Chronic Disease Prevention and Health Promotion, CDC.

Raising the minimum legal sales age (MLSA) for tobacco products to 21 years (T21) is a strategy to help prevent and delay the initiation of tobacco product use (*1*). On December 20, 2019, Congress raised the federal MLSA for tobacco products from 18 to 21 years. Before enactment of the federal T21 law, localities, states, and territories were increasingly adopting their own T21 laws as part of a comprehensive approach to prevent youth initiation of tobacco products, particularly in response to recent increases in use of e-cigarettes among youths (*2*). Nearly all tobacco product use begins during adolescence, and minors have cited social sources such as older peers and siblings as a common source of access to tobacco products (*1*,*3*). State and territorial T21 laws vary widely and can include provisions that might not benefit the public’s health, including penalties to youths for purchase, use, or possession of tobacco products; exemptions for military populations; phase-in periods; and preemption of local laws. To understand the landscape of U.S. state and territorial T21 laws before enactment of the federal law, CDC assessed state and territorial laws prohibiting sales of all tobacco products to persons aged <21 years. As of December 20, 2019, 19 states, the District of Columbia (DC), Guam, and Palau had enacted T21 laws, including 13 enacted in 2019. Compared with T21 laws enacted during 2013–2018, more laws enacted in 2019 have purchase, use, or possession penalties; military exemptions; phase-in periods of 1 year or more; and preemption of local laws related to tobacco product sales. T21 laws could help prevent and reduce youth tobacco product use when implemented as part of a comprehensive approach that includes evidence-based, population-based tobacco control strategies such as smoke-free laws and pricing strategies (*1*,*4*).

Information regarding T21 laws enacted as of December 20, 2019, was obtained from the CDC State Tobacco Activities Tracking and Evaluation (STATE) System for the 50 states, DC, American Samoa, Guam, Marshall Islands, the Commonwealth of the Northern Mariana Islands, Palau, Puerto Rico, and the U.S. Virgin Islands.* Legislation information collected quarterly from the Lexis online legal research database is analyzed, coded, and entered into STATE by CDC. Provisions of T21 laws assessed in STATE include purchase, use, or possession penalties; entities responsible for enforcement; and enacted and effective dates.^†^ In addition, T21 laws were examined to ascertain the inclusion or exemption of military populations; and state T21 and licensing statutes were assessed to determine whether the state prohibits localities from enacting laws to address retail tobacco product sales (i.e., “preemption”). Using STATE, cigarette tax rates (per pack of 20 cigarettes) and comprehensive smoke-free laws prohibiting smoking in all indoor areas of worksites, restaurants, and bars were also assessed as an indicator of the state’s current implementation of evidence-based tobacco control strategies (*4*).

The number of state-level jurisdictions with T21 laws increased from one (Palau) in 2013 to 22 (including 19 states, DC, Guam, and Palau) as of December 20, 2019 (Figure). Six states, DC, Guam, and Palau enacted T21 laws during 2013–2018, and 13 states enacted T21 laws in 2019 (Table). Compared with 2013–2018, more laws enacted during 2019 contained purchase, use, or possession penalties (six versus nine, respectively); military exemptions (one versus six, respectively); or phase-in periods of 1 year or more (two versus four, respectively). Six states with T21 laws enacted in 2019 preempt local laws to address tobacco product sales, compared with four with such laws enacted before 2019.

**FIGURE:**
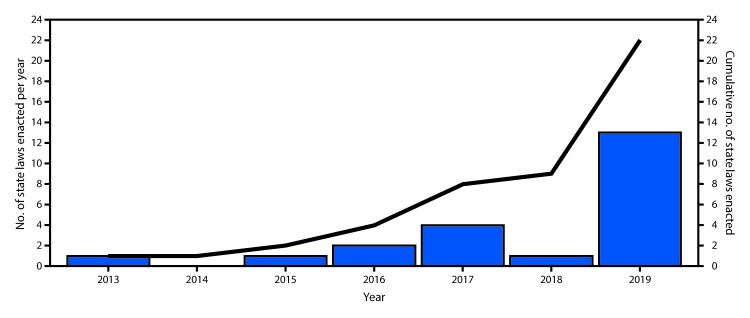
Number of states and territories that have enacted laws prohibiting sales of tobacco products to persons aged <21 years — United States, 2013–2019

**TABLE:** Provisions of state and territorial laws prohibiting tobacco sales to persons aged <21 years (T21) — United States, December 20, 2019

Jurisdiction	Provisions of T21 laws						Preemption of local laws that address retail sales of tobacco products^†^	Comprehensive smoke-free law	Cigarette tax rate ($/pack)
	Penalize youths for purchase, use, and/or possession	Potential youth penalties	Exemption for members of the armed services	Entity responsible for enforcement*	Enacted date	Effective date			
**Arkansas**	Purchase, Use, Possession	Community service and educational program; Fine	Yes	Substance control board	3/28/2019	12/31/2021	Prohibits MLSA >21,**^†^** Other local retail policies	No	1.15
**California**	Purchase, Possession	Fine or community service	Yes	Health entity	5/4/2016	6/9/2016	Prohibits MLSA >21	Yes (includes e-cigarettes)	2.87
**Connecticut**	None	None	No	Financial entity	6/18/2019	10/1/2019	None	No	4.35
**Delaware**	None	None	No	Law enforcement	4/17/2019	7/16/2019	Prohibits MLSA >21, Other local retail policies	Yes (includes e-cigarettes)	2.10
**District of Columbia**	Purchase, Possession	Civil penalty	No	None explicitly listed	11/29/2016	11/29/2016	N/A	Yes (includes e-cigarettes)	4.94
**Guam**	Purchase, Use, Possession	Educational program	No	Health entity; Financial entity	3/23/2017	1/1/2018	N/A	No	4.00
**Hawaii**	Purchase, Use, Possession	Fine	No	None explicitly listed	6/19/2015	1/1/2016	Prohibits MLSA >21, Other retail policies	Yes (includes e-cigarettes)	3.20
**Illinois**	Purchase	Petty offense	No	Law enforcement	4/8/2019	7/1/2019	None	Yes	2.98
**Maine**	Purchase	Civil penalty; educational program	No	Law enforcement	8/2/2017	7/1/2021	None	Yes	2.00
**Maryland**	None	None	Yes	Health entity	5/13/2019	10/1/2019	Inconclusive	Yes	2.00
**Massachusetts**	None	None	No	None explicitly listed	7/27/2018	12/31/2021	Prohibits MLSA >21	Yes (includes e-cigarettes)	3.51
**New Jersey**	None	None	No	Health entity, Law enforcement	7/21/2017	11/1/2017	None	Yes (includes e-cigarettes)	2.70
**New York**	None	None	No	Health entity, Law enforcement	7/16/2019	11/13/2019	None	Yes (includes e-cigarettes)	4.35
**Ohio**	Purchase, Use, Possession	Community service	No	None explicitly listed	7/18/2019	10/17/2022	None	Yes	1.60
**Oregon**	Purchase, Possession	Misdemeanor	No	Health entity	8/9/2017	1/1/2018	Prohibits other local retail policies	Yes (includes e-cigarettes)	1.33
**Palau**	None	None	No	Law enforcement	5/27/2013	6/6/2013	N/A	No	5.00
**Pennsylvania**	Purchase	Summary offense; community service, cessation program, and/or a fine	Yes	Health entity, Substance control board	11/27/2019	07/01/2020	Prohibits MLSA >21, Other local retail policies	No	2.60
**Texas**	Purchase, Use, Possession	Fine; educational program or community service	Yes	Law enforcement, Financial entity	6/7/2019	8/31/2022	Prohibits MLSA >21, Other local retail policies	No	1.41
**Utah**	Purchase, Possession	Misdemeanor; fine and educational program	Yes; also exempts spouses and dependents aged ≥19 yrs	Health entity	3/25/2019	7/1/2021	Prohibits MLSA >21, Other local retail policies	Yes (includes e-cigarettes)	1.70
**Vermont**	Purchase, Possession	Fine or community service	No	Substance control board	5/16/2019	9/1/2019	None	Yes (includes e-cigarettes)	3.08
**Virginia**	Purchase, Possession	Civil penalty; fine or community service	Yes	Law enforcement, Substance control board	2/21/2019	7/1/2019	Inconclusive	No	0.30
**Washington**	Purchase, Possession	Civil penalty; fine and/or community service	No	Substance control board, Law enforcement	4/5/2019	1/1/2020	Prohibits MLSA >21, Other local retail policies	Yes	3.03
**Total**	**None: 6 states and Palau**	**None: 6 states and Palau**	**No exemption: 12 states, DC, Guam, Palau**	**Law enforcement: 8 states, Palau; Health entity: 7 states, Guam; Substance control board: 5 states; Financial entity: 2 states, Guam; None specified: 3 states, DC**	**2013: Palau; 2015: 1 state; 2016: 1 state, DC; 2017: 3 states, Guam; 2018: 1 state; 2019: 13 states**	**Phase-in period of <1 year: 13 states, DC, Guam, Palau**	**No preemption: 7 states**	**Yes, includes e-cigarettes: 9 states, DC; Yes, smoking only: 5 states**	**>3.00: 6 states, DC, Guam, Palau**

**Abbreviation**: MLSA = minimum legal sales age.

* “Law enforcement” includes law enforcement officers (Maine, New Jersey, New York, Texas, and Virginia), Department of Safety and Homeland Security (Delaware), State's Attorney (Illinois), Bureau of Public Safety (Palau), and peace officers (Washington); “Health entity” includes state and local health departments (California, Maryland, New Jersey, New York, Oregon, and Utah) and Departments of Mental Health and Substance Abuse (Guam); “Substance control board” includes Tobacco Control Board (Arkansas), Tobacco Settlement Act contractor (Pennsylvania), Board of Liquor and Lottery (Vermont), Alcohol Beverage Control Agents (Virginia), and State Liquor and Cannabis Board (Washington); “Finance entity” includes Commissioner of Revenue Services (Connecticut), Director of Revenue and Taxation (Guam), and Comptroller (Texas).

^†^ State T21 and licensing statutes were assessed to determine whether local laws that address retail tobacco product sales are preempted. “Prohibits MLSA >21” indicates states that prohibit localities from raising the MLSA for tobacco products above age 21 years; “Other local retail policies” indicates states that preempt local regulations of tobacco product sales and distribution other than minimum sales age, including retailer licensing (Arkansas, Delaware, Hawaii, Pennsylvania, Utah, and Washington), preemption of local retailer licensing (Texas), and preemption of local vending machine regulations (Oregon); “Inconclusive” indicates states with no explicit preemption, but unclear scope of local authority based on case law; and “N/A” includes District of Columbia and territories.

Compared with 2013–2018, more T21 laws enacted in 2019 occurred in states and territories without comprehensive smoke-free laws (two versus five, respectively), or with a cigarette tax rate of <$2.00 per pack (one versus five, respectively). Among the 19 states, DC, Guam, and Palau that had T21 laws as of December 2019, 13 states, DC, and Guam impose a range of penalties for youths, whereas six states and Palau do not impose penalties for youths. Similarly, T21 laws place responsibility for enforcement on a range of entities, most commonly law enforcement (eight states and Palau) and health entities (seven states and Guam).

## Discussion

Before Congress enacted a federal T21 law in December 2019, 19 states, DC, Guam, and Palau had enacted T21 laws. Provisions contained within these state and territorial T21 laws varied widely, and many of the more recently enacted laws contain provisions that could minimize their public health impact. For example, research indicates that purchase, use, or possession penalties might not be effective for changing behavior (*5*), and African-American and Hispanic youths are more likely to be cited for violations than their peers (*6*). In addition, military exemptions exclude young adults at risk for tobacco product use and limit the population-level impact of T21 laws (*7*), and preemption impedes localities from implementing future evidence-based tobacco control strategies (*4*,*8*). In contrast, the federal T21 law, which became effective immediately upon passage, does not include purchase, use, or possession penalties or military exemptions. Adequately enforced T21 policies, coupled with evidence-based tobacco control strategies, such as comprehensive smoke-free laws and high prices for tobacco products, can help prevent and reduce youth use of tobacco products (*1*,*4*,*8*).

A well-enforced, nationwide T21 law has been projected to result in a 12% decrease in tobacco product use prevalence and to avert 223,000 premature deaths (*1*). However, the impact of recently enacted state T21 laws is still being evaluated, and a 2015 Institute of Medicine report noted that “evidence on the independent effect of youth access policies in the context of other tobacco control policies is mixed” (*1*). T21 laws are therefore complements to, but not substitutes for, other evidence-based tobacco control strategies (*1*,*8*). However, despite evidence that population-level strategies such as comprehensive smoke-free laws and high prices for tobacco products prevent youth tobacco product use (*4*,*8*), of the 19 states, DC, and two territories with T21 laws, five states, Guam, and Palau do not have comprehensive smoke-free laws and six states have cigarette tax rates below the national average (mean) tax per pack.

Similar to the federal T21 law, most state T21 laws do not exempt military populations or feature extended phase-in periods. In 2015, Hawaii became the first state to enact a T21 law that included military populations, which was implemented within 7 months. Given that tobacco product use adversely impacts military readiness, T21 laws without military exemptions help promote both public health and national security goals. In 2019, the surgeons general of the Air Force, Army, Navy, and United States wrote a joint letter recommending that Department of Defense leadership make tobacco product use less convenient and coordinate with local, state, and national efforts to reduce tobacco product use.^§^

In recent years, the tobacco industry, including e-cigarette manufacturers, has voiced public support for T21 laws (*9*). However, industry-sponsored tobacco control laws have historically featured provisions that undermine youth tobacco prevention goals (*8*). In 2012, the U.S. Surgeon General noted that tobacco industry–supported youth access bills tend to include provisions that preempt stricter local laws; place responsibility for enforcement on agencies without necessary capabilities; complicate prosecution of retailers for violations; and focus penalties on youths for tobacco product purchase, use, or possession (*8*). In 2014, the Surgeon General concluded that the epidemic of tobacco use was initiated and has been sustained by the tobacco industry (*4*). Therefore, it is important to closely monitor provisions within industry-supported strategies and to assess their potential to adversely affect public health objectives.

The findings in this report are subject to at least one limitation. STATE does not account for local laws, bills under consideration, regulations, or opinions of attorneys general, and it does not systematically account for case law decisions. For example, at least 470 localities have enacted T21 laws, beginning in 2005 onward, many in states that subsequently enacted statewide T21 laws (*10*). The federal T21 law that went into effect on December 20, 2019, applies to the sale of all tobacco products to all persons aged <21 years throughout the United States, its territories, and on tribal lands. Enforcement of the law is focused on retailer, rather than youth, compliance. The federal T21 law does not preempt more stringent state, local, territorial, or tribal MLSA laws, nor limit state or local authority to regulate the sale of tobacco products; however, if those laws are not as strong as federal law, retailers still must comply with the federal law. Jurisdictions may continue to adopt their own T21 laws to help bolster compliance or may raise the MLSA to >21 years.^¶^ The law requires the Food and Drug Administration to publish a final rule updating the current age of sale regulations within 180 days.

Even though youth cigarette smoking has been steadily declining for 2 decades, overall tobacco product use among youths has increased in recent years, driven primarily by unprecedented increases in current e-cigarette use (*2*). A comprehensive strategy that combines evidence-based strategies, such as comprehensive smoke-free laws and pricing strategies, as well as newer strategies such as T21 laws, can help prevent and reduce tobacco product use among U.S. youths (*1*,*4*).

**Summary**

**What is already known about this topic?**

Raising the minimum legal sales age for tobacco products to 21 years (T21) is a strategy to help prevent and delay the initiation of tobacco product use.

**What is added by this report?**

Before Congress enacted a federal T21 law on December 20, 2019, 19 states, DC, and two territories had enacted T21 laws, including 13 in 2019. Several state and territorial T21 laws include penalties for youth purchase, use, or possession of tobacco products; military exemptions; phase-in periods; or preemption of local tobacco retail laws.

**What are the implications for public health practice?**

A strategy combining comprehensive smoke-free laws, pricing strategies, and T21 laws free of purchase, use, or possession penalties, preemption, or military exemptions, can help prevent and reduce youth tobacco product use.

## Footnotes

*
https://www.cdc.gov/STATESystem/ .

† Information is also available in STATE regarding penalties to retailers for violations of minimum legal age of sale laws.

§
https://www.stripes.com/opinion/tobacco-product-use-threatens-military-readiness-1.589063 .

¶
https://www.fda.gov/tobacco-products/retail-sales-tobacco-products/selling-tobacco-products-retail-stores .
